# Subcutaneous Connective Tissue Reactions to Various Endodontic Biomaterials: An Animal Study

**DOI:** 10.5681/joddd.2013.003

**Published:** 2013-02-21

**Authors:** Mohammad Ali Saghiri, Nader Tanideh, Franklin Garcia-Godoy, Mehrdad Lotfi, Kasra Karamifar, Dariush Amanat

**Affiliations:** ^1^Head, Center for Excellence in Endodontic Materials, Professor Kamal Asgar Research Center (KARC) For Dental Materials and Devices, Tehran, Iran; ^2^Assistant Professor, Stem Cell and Transgenic Technology Research Center, Department of Pharmacology, Shiraz Medical School, Shiraz University of Medical Sciences, Shiraz, Iran; ^3^Professor, Bioscience Research Center, College of Dentistry, the University of Tennessee Health Science Center, Memphis, TN, USA; ^4^Professor, Research Center for Pharmaceutical Nanotechnology and Department of Endodontics, Faculty of Dentistry, Tabriz University of Medical Sciences, Tabriz, Iran; ^5^Private Practice, Shiraz, Iran; ^6^Associate Professor, Department of Oral Medicine, Faculty of Dentistry, Shiraz University of Medical Sciences, Shiraz, Iran

**Keywords:** Bioaggregate, biocompatibility, endodontic cement, Geristore, mineral trioxide aggregate

## Abstract

**Background and aims:**

Biocompatibility of root-end filling materials is a matter of debate. The aim of this study was to compare the biocompatibility of a variety of commercial ProRoot WMTA cements and a resin-based cement (Geristore®) with different pH values of setting reaction and different aluminum contents, implanted into the subcutaneous connective tissue of rats at various time intervals.

**Materials and methods:**

Fifty Sprague-Dawley rats were used in this study. Polyethylene tubes were filled with Angelus WMTA, ProRoot WMTA, Bioaggregate, and Geristore. Empty control tubes were implanted into subcutaneous tissues and harvested at 7-, 14-, 28- and 60-day intervals. Tissue sections of 5 μm were stained with hematoxylin and eosin and observed under a light microscope. Inflammatory reactions were categorized as 0, none (without inflammatory cells); 1, mild (inflammatory cells ≤25); 2, moderate (25–125 inflammatory cells); and 3, severe (>125 inflammatory cells). Statistical analysis was performed with Kruskal-Wallis and Mann Whitney U tests.

**Results:**

ProRoot WMTA and Angelus elicited significantly less inflammation than other materials (P<0.05). After 7 days, however, all the materials induced significantly more inflammation than the controls (P<0.05). Angelus-MTA group exhi-bited no significant differences from the Bioaggregate group (P=0.15); however, ProRoot WMTA elicited significantly less inflammation than Bioaggregate (P=0.02). Geristore induced significantly more inflammation than other groups (P<0.05).

**Conclusion:**

Geristore induced an inflammatory response higher than ProRoot WMTA; therefore, it is not recommended for clinical use.

## Introduction

Mineral trioxide aggregate (MTA) is a hydraulic silicate cement (HSC), with several advantages including sealing, sterilizing, mineralizing, dentinogenic and osteogenic capacities.^1^ HSC is composed of tricalcium silicate, dicalcium silicate, tricalcium aluminate, tetracalcium aluminoferrite, and calcium sulfate.^1^ Almost all these cements contain some trace elements like aluminum.^2^ However, incorporation of aluminum is not suitable for biomedical purposes.^3^ Numerous studies have evaluated the biological and physical characteristics of MTA, such as setting time,^1^ acidic resistance,^4^ push-out bond strength,^5^ porosity,^6^ neurotoxicity,^7^ sealing ability,^8^ and the effect of environmental conditions on biocompatibility of MTA.^9^ MTA has been demonstrated to be non-toxic toward living tissues in many investigations in spite of aluminum as one of its components.^10-12^

Bioaggregate (BA) (Innovative Bioceramix, Vancouver, BC, Canada), a white nanoparticle-sized ceramic cement is composed of calcium silicate, calcium hydroxide, and hydroxyapatite and is used as a root-end filling material.^13^ BA has displayed cytocompatibility similar to MTA.^13,14^

Geristore (Den-Mat, Santa Maria, CA) is a hydrophilic Bis-GMA.^15-17^ Geristore is able to bond in the presence of moisture. Its histological biocompatibility, adherence to dentin and cementum, release of fluoride, lack of microleakage, low coefficient of thermal expansion and low polymerization shrinkage all combine to make it the restoration of choice for subgingival restorations when there is the possibility of trans-gingival contamination with the saliva.^18,19^

This study was designed to compare the biocompatibility of three types of hydraulic cement-based materials, including ProRoot WMTA, Angelus WMTA and BA with a resin-based cement, Geristore, in the subcutaneous connective tissue of rats at 7-, 14-, 28-, and 60-day intervals.

## Materials and Methods

The research protocol was approved by the Research Ethics Committee of Shiraz University of Medical Sciences. All the experiments were carried out in accordance to the rules of Institutional Animal Care and Use Committee (IACUC). The method used in this study was similar to those used previously.^11,12^Fifty 3-month-old male Sprague-Dawley rats weighting 220±20 g were randomly used in this study. The animals were kept in a restricted access room under controlled temperature (22°C) and light/dark cycles (12h/12h) and with free access to food and water (ad libitum); each cage housed three rats. All the animals were randomly divided into 5 groups (n=10) as follows:

1. ProRoot WMTA (Tooth-colored Formula; Dentsply, Tulsa Dental, Tulsa, OK, USA)
2. Angelus WMTA (Tooth-colored Formula Angelus, Londrina, Brazil)
3. Geristore (Den-Mat Corporation, Santa Maria, CA)
4. Bioaggregate (Innovative Bioceramix, Vancouver, BC, Canada)
5. Control group (Polyethylene tubes)

Each material was mixed according to manufacturers’ instructions under aseptic conditions. All the operations were performed under general anesthesia by intramuscular injection of 10% ketamine hydrochloride (90 mg/kg, IM, Alfasan Nederland BV, Woerden, The Netherlands) and 2% xylazine (8 mg, IM. Alfasan Nederland BV, Woerden, The Netherlands). Three separate 2-cm incisions were made on the back of the rats at least 2 cm away from each other. Freshly mixed cements were prepared and placed in sterile polyethylene tubes measuring 1.1 mm in inner diameter and 8 mm in length and were immediately implanted subcutaneously into two separate incisions. An empty polyethylene tube was implanted as a control. All the samples were harvested at 7-, 14-, 28- and 60-day intervals. The rats were euthanized by carbon dioxide inhalation with subsequent exsanguination.^20^

The tubes and surrounding tissues were removed in blocks and fixed in 10% buffered formalin solution for 2 weeks; 5-μm tissue sections were prepared longitudinally through the midline of the tubes and stained with hematoxylin and eosin. Evaluations of inflammatory cells (lymphocytes, plasmocytes, polymorphonuclear leukocytes, macrophages, and giant cells) were carried out in microscopic fields adjacent to the test materials at the end of the tubes under a light microscope (Carl Zeiss, Oberkochen, Germany) at ×400 magnification. An average value for each specimen was obtained from the sum of cells counted in 4 separate areas.^21-23^ The observer did not have any knowledge of the materials used in the specimens. The overall mean value for each material was determined in subjects at each time interval. The inflammatory reactions were categorized as:

- 0: none (without inflammatory cells)
- 1: mild (<25 inflammatory cells)
- 2: moderate (25–125 inflammatory cells)
- 3: severe (>125 inflammatory cells)

### Statistics

Kruskal-Wallis and Mann-Whitney U tests were used for statistical analysis. Statistical significance was defined at P < 0.05.

## Results

### 7 and 14 Days

The mean ± SD for Geristore, ProRoot WMTA, Angelus, Bioaggregate, and control groups were 2.90±0.31, 2.40±0.51, 2.50±0.52, 3.00±0.00, and 1.20±0.42, respectively. ProRoot WMTA and Angelus-MTA elicited significantly less inflammation than other materials (P < 0.05). However, all the materials induced significantly more inflammation than the control group (P < 0.05).

### 28 Days

The mean ± SD for Geristore, ProRoot WMTA, Angelus-MTA, BA, and control groups were 2.50 ± 0.52, 2.00 ± 0.00, 2.20 ± 0.42, 2.38 ± 0.51, and 1.20 ± 0.42, respectively. Geristore, and BA elicited significantly more inflammation than ProRoot WMTA and Angelus-MTA groups (P < 0.05). However, all the materials showed significantly more inflammation than the control group (P < 0.05).

### 60 Days

**Figure 1:**
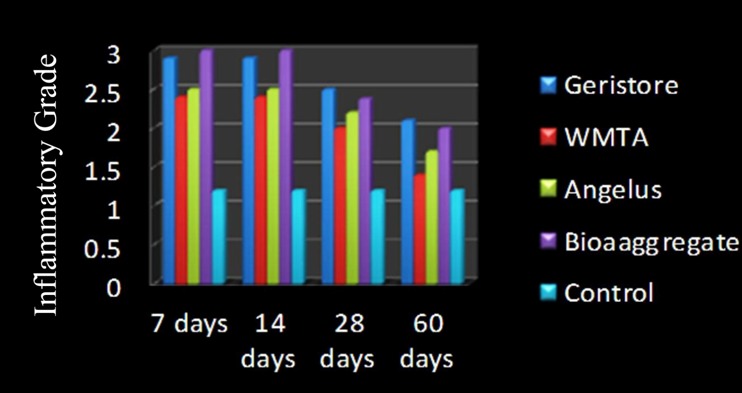
The means of inflammatory grades for Geristore, PROROOT WMTA, Angelus, BA, and control groups after dorsal implantation of test materials at different intervals in rats.

**Figure 2:**
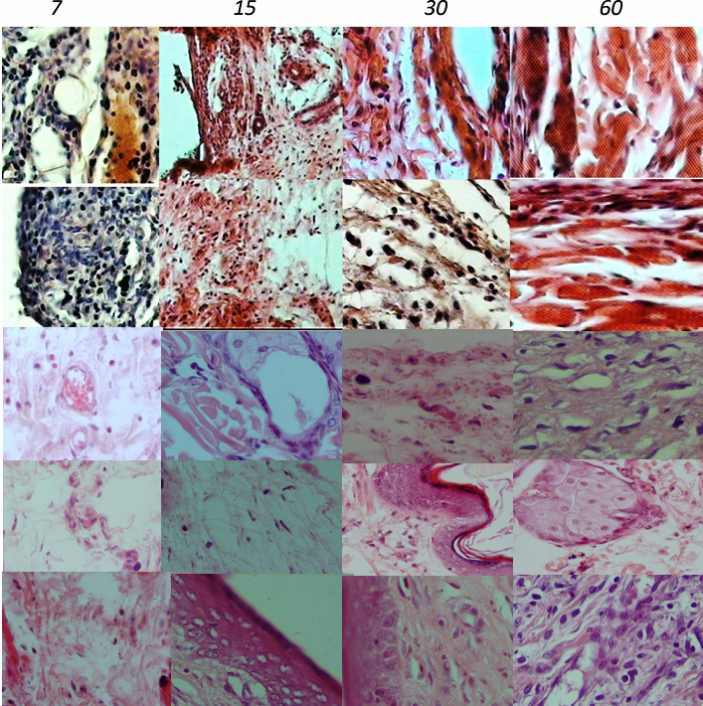
Histological images of inflammatory cell infiltration at the end of implanted tubes in both groups (First row = ProRoot WMTA; Second row = Angelus WMTA; Third row = Bioaggregate; Fourth row = Geristore® and Fifth row = Control Group) (hematoxylin/eosin staining; original magnification, ×400). Exposure time had inverse effects on the inflammatory response.

The mean ± SD for Geristore, ProRoot WMTA, Angelus-MTA, BA, and control groups were 2.10±0.56, 1.40±0.51, 1.70±0.48, 2.00±0.00, and 1.20±0.42, respectively. There were no significant differences between the ProRoot WMTA and Angelus-MTA groups (P=0.18). Moreover, Angelus-MTA group did not exhibit any significant difference from the BA group (P=0.15); however, ProRoot WMTA elicited significantly less inflammation than BA (P=0.02) (Figure 2). There were no significant differences between the control and WMTA or WMTA Angelus-MTA groups (P > 0.05). However, there were significant differences between either the Geristore group or the Bioaggregate group and the control group (P < 0.05). In other words, Geristore and bioaggregate induced more inflammation even after 60 days (Figure 1).

## Discussion

Subcutaneous implantation was used to evaluate the biocompatibility of biomaterials. This technique was introduced by Torneck^23^ in 1966and confirmed by Olsson et al^15^ in 1981.

The differences between the experimental and the control groups at 7-day interval were significant, consistent with the results of other studies.^11,12^ At 7-day interval, both MTA-based cements evoked moderate to severe inflammatory reactions. These results are partially consistent with the findings of a previous study,^12^ showing moderate subcutaneous reaction to three types of MTA.

At 14-day interval, ProRoot WMTA provoked moderate to severe inflammatory reactions. In a similar manner, the results of some studies have shown moderate to severe reactions to ProRoot WMTA.^11,12^ The main major phase of MTA cements, C3S (3CaO, SiO_2_), C2S (2CaO, SiO_2_), and C3A (3CaO, Al_2_O_3_), might be influenced by the pH of surrounding materials or their ingredients, impeding or improving the biocompatibility of the cement.^16,17^ Some studies have shown that ProRoot WMTA actively promotes hard tissue formation by inducing osteogenesis and cementogenesis. MTA cement interacts with the surrounding tissues and forms a shell on the surface, which improves osteogenesis.^17,24^

At 28- and 60-day intervals, all the groups, experimental and control, exhibited mild to moderate infiltration of inflammatory cells. Similarly, an investigation revealed mild to moderate inflammatory reactions to 30-day- and 60-day-old subcutaneous ProRoot WMTA and GMTA.^12^ When MTA powder is mixed with water, calcium phosphate and calcium oxide are released. When ProRoot WMTA comes in contact with tissue fluids, it produces calcium hydroxide.^25^ A high pH and release of calcium and phosphorus ions are required for a material to stimulate mineralization during the process of hard tissue healing.^26^

Geristore has a low pH compared to Bioaggregate, which has a higher pH after the setting reaction. Previous studies have confirmed that both acidic and alkaline conditions influence inflammatory cells through different routes and the present study illu illustrated the effect of both alkaline and acidic materials on inflammation severity.^5-12^ The excellent biocompatibility of a hydraulic cement-based material and other calcium-containing materials might be attributed to their ability to release calcium ions which react with phosphate ions of tissue fluids, resulting in hard tissue formation.^27^ Furthermore, high pH levels contributes to the antibacterial activity which is a critical factor in the formation of a mineralized tissue barrier.^28^

According to manufacturer’s claims, the hybrid ionomer-composite materials, such as Geristore, with the same indications as MTA-based cements, are biocompatible. Geristore was selected due to its different setting pH value compared to other cements. Geristore has a low pH after setting reaction, which might explain the induction of significantly more inflammation than other groups even after 60 days.^18,19^ Geristore has some advantages such as insolubility in oral fluids, increased adhesion to tooth structure, dual-curing capabilities, low polymerization shrinkage, low coefficient of thermal expansion, radiopacity, fluoride release, and biocompatibility.^18^ Geristore has been reported to be less biocompatible than gray MTA,^29^ consistent with the results of the present study. Geristore releases five monomers of Bis-GMA, Bis-DMA, TEGDMA, UDMA and Bisphenol A (Table 1). Furthermore, Geristore releases calcium, and aluminum ions, and fluoride.^30^ Resin monomers are reported to show cytotoxic effects ^31,32^ and might be capable of tumor initiation at relatively low concentrations.^33^

**Table 1:** Main composition of the test materials in each group

Test materials	Composition
ProRoot WMTA	tricalcium silicate, dicalcium silicate, tricalcium aluminate and tetracalcium aluminoferrite
Angelus WMTA	tricalcium silicate, dicalcium silicate, tricalcium aluminate and tetracalcium aluminoferrite
Bioaggregate®	tricalcium silicate, dicalcium silicate, tantalum pentoxide, and calcium phosphate monobasic.Tantalum pentoxide
Geristore®	Bis-GMA, TEGDMA, UDMA, Bis-DMA, and Bisphenol A

Formation of calcium hydroxide is the cause of high alkalinity of MTA after hydration,^34^ which is considered an initial tissue irritant when ProRoot WMTA comes into contact with the tissue.^35^ This would explain the inflammatory reactions subsequent to the subcutaneous implantation of ProRoot WMTA.^36^

Although a previous study has shown that hydraulic silicate-based cement has good market in endodontics, the present study confirmed that inflammation in the BioAggregate group was more severe or equal to the Geristore group, especially at 7-, 14-, and 28-day intervals; therefore, it should be noted that although Bioaggregate is a silicate-based cement, lack of trace elements, such as aluminum, might accelerate setting and/or hydration reaction of this kind of cement, with an important role in its hydration.^1^

The presence of aluminum is a major disadvantage of the materials derived from Portland cement (such as MTA) when used for biomedical and dental applications.^37^ Aluminum ions are released into human biological systems during hydration and setting reactions of such cements.^38^ Moreover, in the case of permanent and long-term applications, such as root-end filling and direct pulp capping, tricalcium aluminate in the cements continually releases aluminum ions into the human biological systems.^39^ Aluminum ions are toxic to the human biological systems ^37^ and to osteoblasts,^40^ inhibiting mineralization of bone.^41^

Accumulation of aluminum in the body tends to occur when the gastrointestinal barrier is circumvented, as is the case with implants or dental procedures.^42-44^ Metal oxides, such as aluminum and iron oxides, have been known to cause abnormal tissue reactions equivalent to a chemical insult.^45^

The three above-mentioned cements are based on (or derived from) Portland cement, and as such rely on aluminum compounds to achieve early strength during setting.^46^ Aluminum might improve the strength and solubility of MTA cement. If aluminum was to be removed from such compositions, the increase in strength would be much slower, rendering the cement useless for its intended applications.^47^

Angelus-MTA was selected due to its difference in its aluminum content from ProRoot. Although Angelus-MTA has more aluminum content than ProRoot MTA,^2^ there were no significant differences in tissue reactions at any time interval. ProRoot MTA has a chemical composition similar to that of Angelus-MTA; however, ProRoot MTA is reported to have slightly higher percentages of bismuth oxide than the other one.^2,48^ BA is composed of tricalcium silicate, dicalcium silicate, tantalum pentoxide and monobasic calcium phosphate. Tantalum pentoxide in BA provides radiopacity instead of bismuth oxide in MTA, and monobasic calcium phosphate in BA adjusts its hydrate setting.^13^ BA was selected due to the absence of aluminum in its chemical composition; BA induced more inflammation at all time intervals. The higher inflammation in the BA group, compared to the MTA group, might be attributed to the effect of aluminum compounds on the insolubility of MTA cement.^49^ In addition, the use of small amounts of MTA for clinical applications limits the release of aluminum into tissue fluids, with a potentially toxic effect.

## Conclusion

The three types of HSC-based cements exhibited biocompatibility; minute amounts of aluminum compounds have less negative effects on the inflammatory cell response. Geristore elicited significantly more inflammation, demonstrating that it is not the material of choice for clinical use.
